# Potential Role of the Inflammasome-Derived Inflammatory Cytokines in Pulmonary Fibrosis

**DOI:** 10.1155/2011/105707

**Published:** 2011-06-02

**Authors:** Rupa Biswas, Melisa Bunderson-Schelvan, Andrij Holian

**Affiliations:** Center for Environmental Health Sciences, The University of Montana, Skaggs Building 274, Missoula, MT 59812, USA

## Abstract

Pulmonary fibrosis is a progressive, disabling disease with mortality rates that appear to be increasing in the western population, including the USA. There are over 140 known causes of pulmonary fibrosis as well as many unknown causes. Treatment options for this disease are limited due to poor understanding of the molecular mechanisms of the disease progression. However, recent progress in inflammasome research has greatly contributed to our understanding of its role in inflammation and fibrosis development. The inflammasome is a multiprotein complex that is an important component of both the innate and adaptive immune systems. Activation of proinflammatory cytokines following inflammasome assembly, such as IL-1**β** and IL-18, has been associated with development of PF. In addition, components of the inflammasome complex itself, such as the adaptor protein ASC have been associated with PF development. Recent evidence suggesting that the fibrotic process can be reversed via blockade of pathways associated with inflammasome activity may provide hope for future drug strategies. In this paper we will give an introduction to pulmonary fibrosis and its known causes. In addition, we will discuss the importance of the inflammasome in the development of pulmonary fibrosis as well as discuss potential future treatment options.

## 1. Introduction

Five million people worldwide are affected by pulmonary fibrosis (PF). According to the Pulmonary Fibrosis Foundation and Centre for Clinical Epidemiology and Biostatistics, there is an estimated 200,000 patients with PF of which more than 40,000 die each year in the USA alone [1]. The symptoms of PF are obscure and mimic other clinical pathologies such as chronic obstructive pulmonary disease, heart failure, and aging, thus misdiagnosis can occur. As a consequence, the actual number of people affected by PF may be significantly higher than the reported figure. Furthermore, varying terminology and lack of standard diagnostic criteria have complicated collection of accurate data. 

The average age at the time of diagnosis of PF is 60–65 years. However, in the USA mortality rates in younger populations are increasing. In recent years, the rate of diagnosis for PF has continued to rise in the older population and evidence suggests that mortality rates for both men and women with PF will continue to rise for the foreseeable future [2]. Many people with the disease live only three to five years after diagnosis, as there is no effective treatment for PF. 

Most cases of PF are idiopathic in nature with unknown events initiating the onset of the disease, although various risk factors have been identified. Importantly, PF is not a guaranteed outcome for every individual who is potentially at high risk—suggesting a multifactorial mechanism is involved in development of PF. One hypothesis suggests that key events in the development of PF include an acute inflammatory response following exposure to an injurious agent followed by abnormal tissue repair and disruption of the lung architecture [3]. Alternatively, it has been suggested that the idiopathic form of PF is largely an epithelial-dependent fibrotic process that functions independently of any inflammatory pathways [4]. This theory, along with a lack of efficacy for anti-inflammatory/immunosuppressive therapies in PF patients, has lead to a reevaluation of the role of inflammation in the development of PF [5]. Additionally, evidence in mice suggesting that fibrosis can develop with a minimal amount of inflammation has further complicated the issue [6]. Nevertheless, there is a large body of evidence implicating inflammatory pathways in the overall etiology of PF [7–9]. It is likely that studies exploring more specific arms of the immune system, such as regulation of the inflammasome, will shed new light on its role in the early development of PF.

In contrast to the idiopathic form of PF, there are many known causes such as exposure to respirable particles and toxicants, viral infections, oxidative stress, and gastroesophageal reflux disease that have been shown to initiate an inflammatory response and may induce events associated with PF [10]. Once respired particles and/or pathogens arrive inside the alveoli, they are engulfed by alveolar macrophages, which play an important role in the recognition, uptake, and clearance of particles from the lungs [11]. Normally, uptake of the particle by the macrophage results in phagosome formation. The phagosome can then fuse with the lysosome-releasing lysosomal enzymes and ultimately destroying the foreign matter [12, 13]. However, in some cases, engulfing particles such as silica or asbestos causes lysosomal membrane permeabilization and the release of cathepsins into the cytosol where they contribute to apoptosis signaling [14] and inflammasome activation [15, 16]. At this stage, apoptosis results in incompletely digested particles remaining inside the lung for extended periods of time [17]. Unsuccessful clearance of these particles cause repetitive inflammation due to prolonged interaction with both immune and nonimmune cell populations. Furthermore, the release of cathepsin B activates the inflammasome [18]. 

In addition to environmental exposures, approximately 3% of PF cases are due to adverse side effects of many prescribed drugs [19]. In fact, there are nearly three hundred drugs that have been associated with fibrotic lung diseases, a topic that is thoroughly reviewed by Camus et al. [20]. Unfortunately, most cases of drug-induced PF occur during normal dosing regimens in a small percentage of susceptible individuals, making early detection and prevention difficult [20]. Drugs most commonly associated with irreversible cases of PF include amiodarone, bleomycin, nonsteroidal anti-inflammatory drugs (NSAID), most alkylating agents [20], and methotrexate [21, 22]. Mechanisms of drug-induced PF are mostly unknown; however, evidence suggests that the inflammasome may play a role. For example, bleomycin has been used to induce PF in murine models in order to better understand molecular mechanisms [23]. It has been demonstrated that bleomycin-induced lung inflammation and remodeling is largely mediated by uric acid, which is released from dying cells, upon injury or insult [24]. When uric acid is released from dying cells activation of the NALP3 inflammasome occurs, which results in enhanced IL-1*β* production and subsequent inflammation [24]. In addition, it is believed that bleomycin induces PF pathology through the IL-1R1/MyD88 signaling pathway [25]. Furthermore, a role for the NALP3 inflammasome in acetominophen-induced hepatotoxicity was recently reported and may shed light on the mechanisms of NSAID-induced PF [26]. Lastly, as a folic acid analog, methotrexate is one of the oldest and most efficacious antineoplastic drugs. However, some studies have suggested that a link between the use of methotrexate and development of PF exists [21, 22]. It is likely that the mechanism of methotrexate-induced PF involves modulation of an endogenous anti-inflammatory agent known as adenosine, which has been reported to have profibrotic effects in some experimental models [27] and reportedly activates the NALP3 inflammasome following release from necrotic cells [28].

**Figure 1:**
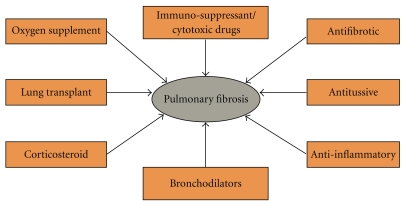
Present treatment options for PF. Available treatment options for PF include antitussives and bronchodilators, which relieve symptoms of dry unproductive cough and bronchospasm, respectively. In addition, corticosteroids are often administered for sustained relief of bronchospasm. Immunosuppressive/cytotoxic agents are usually reserved for patients not responding to steroid treatment or suffering from adverse side effects. Lung transfer surgery is only considered in patients less than 65 years of age and can lead to fatal complications. Oxygen supplementation has positive outcomes on patient health. However, the personal inconvenience may affect patient compliance.

In addition to complications caused by drug-induced PF, little progress has been made toward treatment of the disease. At this time, there is no pharmacological treatment known to restore normal lung tissue architecture, which is paramount to regaining normal lung function [29]. The treatment choices for PF are limited and are aimed at preventing more lung scarring, relieving symptoms and improving the quality of life [30]. Conventional treatment options for PF include antitussives, bronchodilators, corticosteroids, immunosuppressive/cytotoxic agents, and antifibrotic agents [31–33] (see Figure 1). Drugs used to suppress the immune response have been used as a method for treating PF, but like other current therapies, these drugs have had little success in altering the normal course of PF disease progression [31, 34]. However, recent findings on the role of inflammatory pathways and the cytokines involved in those pathways may provide future direction for the development of new drugs for the treatment of PF. Traditionally, onset and progression of PF has been considered an irreversible and progressive disease. However, there is evidence to suggest that under certain conditions, the fibrotic process can be reversed—which has broad implications for PF patients.

Results in our lab demonstrate that collagen deposition, a key step in the fibrotic process, is reversible once exposure to asymmetric dimethylarginine has been removed [35]. In addition, the use of an interleukin-1 receptor (IL-1R) antagonist was able to both prevent and reverse bleomycin-induced pulmonary fibrosis in mice [36]. This suggests that drugs designed against components of the inflammasome may also have potential for future treatment regimens. The inflammasome is an important regulator of the proinflammatory cytokines, interleukin-1 beta (IL-1*β*), and interleukin-18 (IL-18). Both IL-1*β* and IL-18 have been linked to PF development and pathogenesis while clinical and experimental studies point to a crucial role of IL-1*β* and IL-18 in acute and chronic inflammation [37]. Another evidence supporting the potential for the reversal of PF pathology has been demonstrated through studies on IL-13, an inflammatory cytokine known to be involved in fibrosis [38–40]. This has significant implications for PF patients as it provides hope for future treatment strategies focusing on inflammasome activity. 

## 2. Causal Factors in PF Disease Development

### 2.1. Environmental Exposures

While a high percentage of PF cases are idiopathic in nature, PF is also associated with environmental or occupational exposures to respirable asbestos particles, silica, and metal dust. Other common factors that can contribute to PF include a genetic predisposition, some medications, and medical conditions. In addition, exposure to viral infections such as influenza A virus, hepatitis C virus, HIV-A virus, and herpes virus -6 can also increase the risk for PF [10].

### 2.2. Genetics

PF has a complicated etiology that most likely contains a genetic component that is strongly influenced by environmental exposures. Several polymorphisms have been associated with the development of PF. In addition, patients with accelerated clinical progression have different genetic profiles than less severe case studies [41]. 

Cases of familial PF follow an autosomal dominant pattern [42] while the pathological symptoms remain indistinguishable from nonfamilial cases with the exception of the age of onset [43]. However, variable penetrance, even within genetically susceptible individuals and families, suggests that PF disease outcome is highly influenced by environmental factors [42].

## 3. Inflammasome Activity

### 3.1. What Is the Inflammasome?

The inflammasome is an important component of both the innate and adaptive immune systems [44]. The innate immune system provides immediate defense against infections and triggers the T and B cells of the adaptive immune system. Initial studies have identified the NALP (NACHT domain-, leucine-rich repeat and pyrin domain-containing protein) family of proteins as a critical component of the inflammasome [45]. The NALP family of proteins plays a crucial role in alerting the mammalian immune system to the presence of “danger” conditions and pathogens [46]. 

NALP is a member of the NOD-like receptor (NLR) family. Other family members of NLR are NAIP (NLR family, apoptosis inhibitory protein) and NLRC4 (NLR family, CARD domain containing 4). The NLR family members all have similar structures, with a ligand binding leucine-rich repeat domain at the carboxy terminus and an intermediary NACHT domain for nucleotide binding and self-oligomerization [47]. NLR family members also contain an effector domain at the amino terminus consisting of either a pyrin domain (PYD), a caspase activation and recruitment domain (CARD), or a baculovirus inhibitor of apoptosis protein repeat (BIR) [48]. It is thought that the main function of the NLR is the regulation of proinflammatory cytokines such as IL-1*β* and IL-18. Though diverse molecular entities including bacteria, viruses, components of dying cells, immune activators, and crystalline or aggregated materials can activate NLR protein (NLRP), the precise mechanism of how NLRs recognize their ligands is unclear. 

Out of the several NALP proteins, the NALP3, or cryopyrin, inflammasome has been the most extensively studied. Though signals and mechanisms leading to inflammasome activation are still poorly understood, it has been reported that NALP3 activation is required for stimulating inflammasome assembly [48]. Once assembled, the inflammasome activates caspase-1, which catalyzes cleavage of the inactive precursor molecules pro-IL-1*β* and pro-IL-18 to their active forms, IL-1*β* and IL-18 [49]. IL-1*β* and IL-18 can only be secreted out of the cell in their active form [50]. Though caspase-1 is important for cleavage of pro-IL-1*β* and proIL-18, there is evidence that these proinflammatory cytokines can be cleaved by a non-caspase-1 mechanism as well. For example, proteinase-3 has also been reported to cleave pro-IL-1*β* and pro-IL-18 to generate mature forms of these cytokines in the absence of caspase-1 [50]. This may help explain the wide variety of signals that are known to activate the inflammasome.

In addition to NALP, the adaptor protein ASC (apoptosis-associated speck-like protein containing a caspase recruitment domain (CARD)) is an important component of the inflammasome complex. Adaptor protein ASC connects NALP proteins to pro-caspase-1 leading to cleavage of the CARD domain of pro-caspase-1 [46]. It has been reported by several research groups that, in the absence of NALP3 or ASC in genetically deficient mice, there is no activation or maturation of IL-1*β* [24, 51–54]. In fact, the ASC protein is required for bleomycin-induced IL-1*β* production and inflammation [25]. Therefore, NALP3 and ASC are potential therapeutic targets for treating chronic inflammation and fibrosis. 

The exact composition of an inflammasome complex depends on the activator, which initiates inflammasome assembly. For example, NALP-1 can directly bind and activate caspase-1 and/or caspase-5 via its unique CARD domain without the help of the adaptor protein (ASC) [55]. Once the inflammasome complex has been assembled, the downstream signaling due to activation of proinflammatory cytokines can contribute to the progression of many inflammatory diseases, including PF.

### 3.2. Inflammasome Activity and Fibrosis

Molecular mechanisms of acute lung injury resulting in inflammation and fibrosis are not completely understood. However, recent understanding of the inflammasome pathway has lead to recognition for the role of the inflammasome in PF development and pathogenesis [49]. 

Some agents that have been associated with the development of the nonidiopathic forms of PF, such as asbestos and silica, have also been shown to induce lysosomal membrane permeabilization and assembly of the inflammasome [56, 57]. Permeabilization of the lysosomal membrane results in the release of proteases such as cathepsin B into the cytosol [53, 54]. Cathepsin B activates the NALP3 inflammasome and ultimately leads to IL-1*β* and IL-18 cleavage from their proisoforms [52, 58]. In addition, evidence suggests that maturation of IL-1*β* and IL-18 plays a critical role in acute and chronic inflammation, similar to what is observed in cases of PF [37]. For example, a role for IL-1*β* in fibrogenesis has been established using IL-1 receptor deficient mice, possibly through stimulation of the matrix metalloproteinases [59]. In fact, gene expression analysis of patients with histologically proven PF highlighted the importance of matrix metalloproteinase 7, an observation that was supported with studies using matrix metalloproteinase 7 knock-out mice [60]. Similarly, neutralization of IL-18 has been shown to reduce obstruction-induced renal fibrosis [61] and is also known to induce gene expression of IL-1 [44]. IL-1*β*, in particular, is an important mediator of the inflammatory response following injury or infection [62] and may be a therapeutic target for chronic lung inflammation and fibrosis [25]. 

Characteristic changes in proinflammatory cytokine profiles associated with the inflammasome may also result in the recruitment of fibroblasts and inflammatory cells [63]. Fibroblasts and myofibroblasts provide a structural platform for the lung and may be key effectors in PF development. Activation of fibroblasts and myofibroblasts is a pathological hallmark of PF and most likely results from the downstream effects of abnormal cytokine, chemokine, and growth factor activity. In areas with increased fibroblast activity, excessive deposition of extracellular matrix along with loss of the normal structural components of the lung is observed [64].

### 3.3. Downstream Effects of IL-1*β* and IL-18

The proinflammatory cytokines of the IL-1 family, including IL-1*β* and IL-18, play important roles in antimicrobial host defense as well as the modulation of gene expression. Once assembly of the inflammasome and subsequent activation of the cytokines IL-1*β* and IL-18 has occurred via cleavage of their proisoforms, a proinflammatory cascade of events is likely to follow. In vivo, IL-1*β* is primarily responsible for symptoms of acute inflammation such as fever, acute protein synthesis, anorexia, and sleep disturbances [65]. In contrast, IL-18 is largely involved in Th1 responses [44]. In addition, IL-1*β* and IL-18 play a primary role in diseases involving the innate and acquired immune systems such as PF, autoimmunity, rheumatoid arthritis, cancer, metabolic syndrome, and atherosclerosis [66]. 

In renal fibrosis, activation of the inflammasome and subsequent release of IL-1*β*, along with TNF-*α* and IFN-*λ*, contributes to the epithelial mesenchymal transition [67], a critical step in disease development that may have implications for PF. Furthermore, long-term exposure (>10 days) of human epithelioid dermal microvascular endothelial cells to IL-1*β* results in a permanent transformation into a myofibroblast phenotype [68], possibly increasing susceptibility for initiation of the fibrotic process. Further increases in IL-1*β* production will drive a subsequent increase in TGF-*β*, resulting in increased collagen production by fibroblasts—also known to be a critical step in the development of fibrosis [69]. Thus, IL-1*β* and IL-18 most likely contribute to favorable conditions for the development of fibrotic lesions such as increased collagen production and transformation of nonfibroblastic cells into a phenotype more typically seen in fibroblasts or myofibroblasts. It is clear that dysregulation of pathways leading to inflammation and the subsequent development of fibrosis is closely linked to the proinflammatory cytokines IL-1*β* and IL-18, which in turn are dependent on formation of an inflammasome complex for activation.

## 4. Inflammasome Activation

### 4.1. Mineral Particles

Inhalation of mineral particles such as asbestos or silica in environmental or occupational exposures over an extended period of time can result in the non-idiopathic form of PF. Recent studies have demonstrated that silica internalization leads to lysosomal destabilization and NALP3 inflammasome activation [52]. Furthermore, Cassel and colleagues [57] have shown that the NALP3 inflammasome is essential for development of silicosis. In addition, silica exposure activates caspase-1 leading to IL-1*β* maturation and inflammation following lysosomal damage [70]. 

Other mineral fibers such as asbestos lead to an irreversible fibrotic condition known as asbestosis after prolonged exposure. Recent studies have provided clues as to the molecular mechanism by which asbestos leads to pulmonary inflammation and fibrosis. Similar to that seen with silica, asbestos exposure results in activation of the NALP3 inflammasome and IL-1*β* secretion by macrophages [56].

### 4.2. Nanomaterials

Nanomaterials are engineered structures with at least one dimension of 100 nanometers or less and significant potential to be designed in a multitude of shapes and sizes [71]. These materials are increasingly being used for commercial purposes due to their exceptional properties of conductivity, reactivity, and optical sensitivity [71]. Furthermore, modification of nanomaterials resulting in a wire, fiber, belt, or tube is an attractive method for enhancing the end-product usage. However, toxicity and pathological potential can change dramatically when the nanomaterial shape is altered. In particular, if the nanomaterial becomes difficult for a phagocytic cell to process, lysosomal disruption and NALP3 inflammasome activation may occur [72].

## 5. Current and Future Treatment Options For PF

### 5.1. Current Treatment Options in Clinical Trial

Current pharmacological treatment of PF is based on a combination of corticosteroids and immunosuppressants. However, the efficacy of treatment remains a matter of debate and these medications have serious adverse side effects. It is beyond the scope of this paper to include a discussion of all current treatment regimens; however, there are several comprehensive reviews discussing the outcomes of trials aimed at testing standard therapies for PF [73, 74].

### 5.2. New Pharmacological Agents

In recent years, there has been an increase in the launch of new pharmacological agents that are currently in clinical trial stage. Some of these therapeutic agents are interferon gamma, N-acetylcysteine, etanercept (antitumor necrosis factor *α*), bosentan (antiendothelin dual receptor antagonist), and imatinib (tyrosine kinases inhibitor of the PDGF receptor) to name a few. 

**Figure 2:**
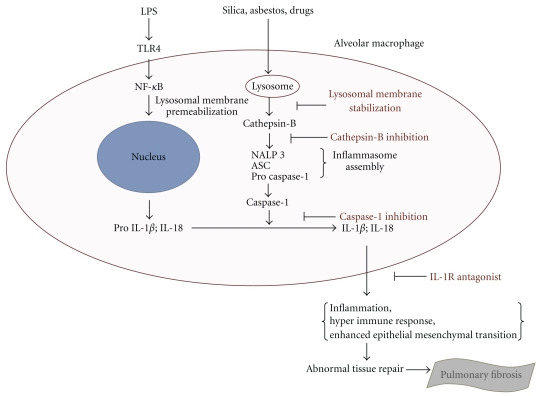
Potential inflammasome targets for novel PF therapeutics. Lysosomal membrane permeabilization plays an important role in cathepsin B release and inflammasome activation. Therefore, stabilization of the lysosomal membrane would prevent initiation of the adverse events associated with inflammasome activation. Similarly, inhibition of cathepsin B would attenuate inflammasome assembly and activation. Further downstream of inflammasome assembly, inhibition of caspase -1, would prevent maturation of proinflammatory cytokines such as IL-1*β* and IL-18. In addition, IL- 1R antagonists would prevent the downstream events following the binding of IL-1*β* to its receptor.

Agents involved in the inflammasome pathway are currently being studied as potential therapeutics for PF as well. For example, the successful use of the IL-1 receptor antagonist, anakinra, to treat bleomycin-induced PF in mice has raised the possibility for its use in human trials [36]. In fact, anakinra is currently being used for the treatment of other inflammatory diseases such as Muckle-Wells Syndrome [75] and rheumatoid arthritis [76]. In addition, inhibition of cathepsin B, an inflammasome activator, has significant potential for therapeutic application in diseases linked to lysosomal dysfunction such as PF [54]. Specifically, Liu et al. [77] reported a mechanism by which the transcription factor NF-*κ*B can inhibit inflammation, cell death, and the subsequent formation of fibrosis through its regulation of the serine protease inhibitor 2A, a potent inhibitor of cathepsin B [78]. Therefore, it is possible that drugs aimed at other components involved in inflammasome activation and activity could have potential to be highly efficacious in reversing or lessening the initial effects associated with the onset of PF (see Figure 2).

## 6. Conclusions

Pulmonary fibrosis is a progressive, disabling, and fatal disease. Present treatment options for PF are inadequate due to an incomplete understanding of the molecular mechanism of the disease progression. Recent research focused on understanding the molecular mechanism of PF development has suggested that inflammasome activation may play a role in initiation and/or progression of the disease. In addition, inflammasome activation is involved in the development of other inflammatory and fibrotic diseases, which may provide clues for our current understanding of PF. Specifically, the NALP3 protein is most likely responsible for inflammasome activation leading to proIL-1*β* and proIL-18 cleavage and maturation by active caspase-1, which contributes to fibrosis development. In fact, evidence suggests that maturation of IL-1*β* and IL-18 plays a critical role in acute and chronic inflammation, similar to what is observed in cases of PF [37], and may result in the recruitment of fibroblasts and inflammatory cells [63]. Fibroblasts and myofibroblasts provide a structural platform for the lung and may be key effectors in PF development. Activation of fibroblasts and myofibroblasts is a pathological hallmark of PF and most likely results from the downstream effects of abnormal cytokine, chemokine, and growth factor activity, such as what is observed following inflammasome activation.

In addition to aberrant regulation of cytokines, the adaptor protein ASC is an important component of the inflammasome complex and is known to play a crucial role in the development of bleomycin-induced PF [25]. Therefore, identifying critical components of the inflammasome complex and its downstream effectors, such as NALP3, ASC, IL-1*β*, and IL-18, may be key for developing potential therapeutic targets, which will be important for novel PF treatments in the future.
